# The moderating and mediating effects of personality on the association between morningness and well-being

**DOI:** 10.7717/peerj.15861

**Published:** 2023-08-11

**Authors:** Soo Jin Lee, Sudong Jeong, Han Chae

**Affiliations:** 1 Department of Psychology, Kyungsung University, Busan, South Korea; 2 School of Korean Medicine, Pusan National University, Yangsan, South Korea

**Keywords:** Morningness, Temperament and character, Well-being, Moderation analysis, Sequential mediation

## Abstract

**Background:**

Morningness (morning-eveningness preference or chronotypes) and personality can be both associated with well-being, but few studies have directly compared these two constructs as correlates of well-being. Thus, the first purpose of this study was to test the effects of interactions between stable personality traits (temperaments) and morningness on well-being. Furthermore, personality factors are often composed of both stable biological factors (temperament) and socio-cultural factors (character), and little is known about personality interplay of temperament and character factors with respect to morningness and well-being. The second purpose of this study was therefore to examine the sequential mediating effects of temperament and character factors on the relationship between morningness and well-being.

**Methods:**

The Composite Scale of Morningness, the Korean version of the Temperament and Character Inventory-Revised Short Version (TCI-RS), and the Satisfaction with Life Scale were used to measure morningness, personality dimensions, and well-being, respectively, in 287 Korean university students. Moderating and sequentially mediating effects of temperament and character traits were determined using Hayes’ PROCESS macro in SPSS after controlling for sex and age.

**Results:**

First, novelty-seeking (NS) and persistence (PS) temperaments have demonstrated the moderating effect in the association between morningness and well-being. The positive effects of morningness on life satisfaction increased with lower NS and PS, respectively. However, other temperaments such as harm avoidance (HA) and reward dependence (RD) have not shown the moderation in the relationship between morningness on well-being. Second, HA temperament and self-directedness (SD) character sequentially mediated the relationship between morningness and well-being. The combination of low scores of HA and high scores of SD have shown the positive effect on the relationship between morningness and well-being.

**Discussion:**

This study demonstrated that both the interactions between temperaments and morningness, and combination of specific TCI-RS temperament and character traits play important roles in influencing the association between morningness and well-being. The significance of the mature SD character and its implications for well-being are discussed with limitation of the present study.

## Introduction

Morningness, or chronotype, exists on a continuum that manifest as individual variation in circadian rhythm (Natale & Cicogna, 2002). The morning-type is located on one extreme of this spectrum, and the evening-type on the other; individuals with morning preferences are active and feel comfortable during the day, whereas their evening-type counterparts are energetic and mobile in the evening or at night. In addition, chronotype has been reported to be associated with many physical diseases, psychiatric disorders, and behavioral problems (Adan et al., 2012; Merikanto et al., 2013).

Many studies have described the relationship between morningness and various individual differences, one of which is subjective well-being (Drezno, Stolarski & Matthews, 2019; Gulec et al., 2013). Morningness has been shown to be positively associated with general well-being and negatively associated with psychopathology (Gulec et al., 2013). There are several perspectives on delineating the associations between morningness and well-being (Bullock, 2019) in the light of genetic, biological, developmental, psychosocial, psychological theories. Further, morningness has a direct influence on subjective well-being, even after controlling for the mediating effects of personality variables (Drezno, Stolarski & Matthews, 2019). This has led us to consider the nature and significance of the relationships between morningness, personality, and well-being.

Extensive research have been conducted on the relationships between morningness and personality factors (Adan et al., 2010; Antunez, Navarro & Adan, 2014; Bullock, 2019; Drezno, Stolarski & Matthews, 2019; Randler & Saliger, 2011) using, for example, Eysenck’s personality dimensions (EPI), the Five Factor Model (FFM), and Cloninger’s Temperament and Character Inventory (TCI). The conscientiousness trait of the FFM consistently demonstrates positive correlations with morningness (Adan et al., 2012). The neuroticism items of the EPI has shown mixed results; however, neuroticism as measured by the FFM is negatively associated with morningness (Adan et al., 2012; Lipnevich et al., 2017). In addition, the novelty-seeking (NS) and harm avoidance (HA) factors of the TCI have shown positive association with eveningness (Lee et al., 2017a). On the other hand, TCI persistence (PS), which is also strongly correlated with FFM Conscientiousness, is reported to exhibit a positive association with morningness (Park et al., 2014). With respect to the relationship between personality and well-being, extensive research has been conducted on the association between personality traits of the FFM and well-being. A meta-analysis (Bullock, 2019; Steel, Schmidt & Shultz, 2008) revealed that well-being is positively and negatively related to extraversion and neuroticism traits, respectively. Furthermore, the conscientiousness and agreeableness factors of the FFM often have a positive relationship with well-being, which seems to align with the characteristics of morning-type individuals (Drezno, Stolarski & Matthews, 2019).

Unlike the well-established linear associations between the FFM personality traits and well-being, the relationship between TCI temperament factors and well-being is somewhat inconsistent: NS and PS have shown mixed results, while HA is primarily negatively correlated and reward dependence (RD) is not correlated with well-being (Garcia, 2011).

These inconsistent results may be due to the interaction of temperament with other variables. Intraindividual personality assumes that individuals develop *via* interactions between the biological being and the environment. It also focuses on the possibility of complex and dynamic systems that exert an effect in the within-person development of personality and well-being (Cervone, 2005). Therefore, the first purpose of this study was to explore the effect of interactions of temperaments (NS, HA, RD, PS) and morningness on the well-being, which could enhance our comprehension of the specific connection among personality, morningness, and well-being. The moderating statistical analysis was used for the interaction analysis of morningness and temperament on well-being. Moderation analysis refers to examination of the statistical interaction between independent variables in predicting a dependent variable (Baron & Kenny, 1986; MacKinnon & Luecken, 2008).

Previous research has chiefly focused on the unchangeable aspects of personality that affect well-being. We should consider the fact that well-being is influenced by innate and biological temperament factors as well as by mature and socio-cultural character dimensions (Cloninger & Zwir, 2018). In Cloninger’s biopsychosocial model of personality (Cloninger, Svrakic & Przybeck, 1993), temperament is people’s tendency to emotionally react automatically and unconsciously in specific ways (*e.g*., joy, sad, anger, disgust, fear). Character, on the other hand, is what people consciously make of themselves intentionally. Accordingly, character regulates the way our temperament makes us react to the internal and external environment (Cloninger, 2004). In addition, Cloninger’s personality model not only includes the non-linear assumption that distinguishes temperament and character but also delineates a more comprehensive and holistic portrait of the individual with interactions of both temperament and character variables. As the concept of the TCI representing biological and socio-cultural factors and their interactions fits here, it could be viewed as the best tool to accurately represent the concept of well-being.

While there are context-dependent advantages and disadvantages of both high and low TCI temperament scores, high character dimension scores are generally regarded as being adaptive; specifically, high scores in the self-directedness (SD), cooperativeness (CO), and/or self-transcendence (ST) items of the TCI are protective against psychopathology and promote mental health (Eley et al., 2013, 2016; Kim, Lee & Lee, 2013).

Within the framework of the TCI, the combination of HA and SD scores often plays an important role in well-being and psychopathology (Chae & Lee, 2022; Cloninger & Zohar, 2011; Lee et al., 2014; Moreira et al., 2015). That is, the combination of low vulnerability (*e.g*., low HA) and high mature-character dimensions (*e.g*., high SD) is associated with increased well-being (Cloninger, 2004; Ryff, Singer & Dienberg Love, 2004). For example, the aggregation of higher scores of HA and lower scores of SD often showed difficulties at work in nurses (Mihailovic et al., 2022). Therefore, the second purpose of the present study is to explore whether the HA temperament and SD character traits sequentially mediate the association between morningness and well-being. The sequential mediating statistical analysis was applied for this purpose. Mediation analysis investigates whether the mediating variables (*e.g*., HA, SD) accounts for a significant amount of shared variance between the independent (*e.g*., morningness) and the dependent variables (*e.g*., well-being).

This study aimed to explain the key roles of the temperament and character dimensions of TCI in biopsychosocial functioning associated with the relationship between morningness and well-being.

## Methods

### Participants

The Composite Scale of Morningness (CSM), TCI-Revised-Short Version (TCI-RS), and Satisfaction With Life Scale (SWLS) were used to measure morningness-eveningness, temperament and character, and life satisfaction, respectively, in a sample of 287 university students in Busan, South Korea. Out of 311 students, 287 (92.3%, 92 men and 195 women) volunteered to participate and completed our questionnaire. Participants were also asked about their age and sex. This study was approved by the Institutional Ethics Board of Kyungsung University, South Korea (approval number KSU-18-06-001-0705) and all participants provided written informed consent.

### Measures

#### Composite scale of morningness

The CSM (Smith, Reilly & Midkiff, 1989) is commonly used to identify the circadian type or morningness. It was translated into Korean by Yoon et al. (1997) and standardized by Kook, Yoon & Lee (1999). The scale consists of 13 items, with three measured on a 5-point Likert scale and 10 on a 4-point scale. The CSM considers morningness-eveningness as a one-dimensional construct, and the total score of the CSM ranges from 16 to 55, with higher scores indicating extreme morningness and lower scores extreme eveningness. A validation study (Kook, Yoon & Lee, 1999) in a sample of Korean nurses and female college students indicated an internal consistency of 0.787–0.836. The internal consistency in this study was 0.85.

#### Temperament and character inventory-revised short version

The TCI-RS consists of two interrelated domains of temperament and character, and is originally developed by Svrakic et al. (1993). The four temperament traits represent biases in automatic responses to emotional stimuli involving involuntary processes, while the three character traits reflect differences in higher cognitive functions of goals, values, and relationships (Cloninger, 2008).

The temperament dimensions comprise NS (impulsiveness, exploratory excitability, extravagance, and disorderliness), HA (fear of uncertainty, anticipatory worry, shyness with strangers, and fatigability), RD (attachment, sentimentality, openness, and dependence), and PS (work hardened, eagerness, ambition, and perfectionism). The character dimensions are SD (purposeful, resourceful, responsible, spontaneous, and self-accepting), CO (helpful, empathic, forgiving, principled, and tolerant), and ST (spiritual, idealistic, and transpersonal).

The TCI-RS Korean version is a 140-item self-report questionnaire which requires individuals to score each item using a 5-point Likert scale. The internal consistencies of NS, HA, RD, PS, SD, CO, and ST were reported to be 0.83, 0.86, 0.81, 0.82, 0.87, 0.76, and 0.90, respectively (Min, Oh & Lee, 2007). The internal consistency in this study could not be provided due to the fact that the TCI-RS is copyrighted and associated data and scoring key are proprietary.

#### Satisfaction with life scale

The SWLS is a five-item self-report questionnaire which measures an individual’s subjective life satisfaction or well-being on a 7-point Likert scale (Diener et al., 1985). The SWLS Korean version was translated by Lee & Lee (2005), and it has an internal consistency of 0.85. The internal consistency of the scale in this study was 0.86.

#### Data analysis

Significant differences between male and female participants in age, morningness, well-being, and TCI-RS personality dimensions were examined using t-tests. Pairwise correlations between morningness, well-being, temperament and character items were determined using Pearson’s correlation, and correlation coefficients were provided.

Two hypotheses were tested: the first (H1) was that NS and PS temperaments would moderate the association between morningness and well-being; the second (H2) was that HA and SD would sequentially mediate the relationship between morningness and well-being. For both hypotheses, moderation and mediation analyses were conducted using Hayes’ PROCESS macro (models 1 and 6, respectively), after controlling for sex and age.

Additionally, a floodlight analysis was conducted (Spiller et al., 2013) using the Johnson-Neyman test in Hayes’ PROCESS macro to understand the moderating effect. The Johnson-Neyman approach tests the effect of the predictor at all levels of the moderator.

The sequential mediation analysis was performed using ordinary least squares regression to estimate direct and indirect effects. Effect size estimates of indirect associations were drawn using bootstrap confidence intervals.

The data are presented as means and standard deviations, and *p* values <0.05 was considered statistically significant. IBM SPSS Statistics 20.0 (IBM, Armonk, NY, USA) was used for all statistical analyses.

## Results

### Demographic features of the study participants

The demographic features of the participants are presented in Table 1. There were significant differences in age (*t* = 2.975, *p* < 0.01), well-being (*t* = 2.654, *p* < 0.01), NS (*t* = 3.419, *p* < 0.01), HA (*t* = −3.161, *p* < 0.01), and ST (*t* = −4.459, *p* < 0.01) but not in CSM (*t* = 0.643, *p* > 0.05), RD (*t* = −1.212, *p* > 0.05), PS (*t* = 1.538, *p* > 0.05), SD (*t* = 1.538, *p* > 0.05), or CO (*t* = 1.538, *p* > 0.05) between men and women. The higher male age is a consequence of men in South Korea often having to complete their mandatory military duty in their lower school years. Considering this finding and the significant sex-related differences identified, we performed all analyses with age and sex as covariates.

**Table 1:** Demographic characteristics of the participants.

Variables	Total	Male	Female	*t*
	(N = 287)	(*n* = 92)	(*n* = 195)	
Age	20.55 (1.629)	21.04 (2.158)	20.32 (1.249)	2.975 **
CSM	30.54 (6.741)	30.91 (6.838)	30.36 (6.705)	0.643
SWLS	20.44 (6.177)	21.84 (6.097)	19.78 (6.12)	2.654**
NS	37.35 (10.104)	40.07 (8.552)	36.07 (10.539)	3.419**
HA	41.13 (11.984)	37.92 (11.554)	42.64 (11.914)	−3.161**
RD	46.35 (10.658)	45.24 (10.368)	46.87 (10.778)	−1.212
PS	42.63 (10.248)	43.01 (10.956)	42.45 (9.920)	0.435
SD	44.25 (11.019)	45.85 (10.594)	43.49 (11.160)	1.696
CO	57.26 (9.794)	56.99 (9.512)	57.38 (9.946)	−0.319
ST	23.19 (11.309)	19.25 (9.561)	25.05 (11.610)	−4.469***

**Notes:**

**p < 0.01; ***p < 0.001.

CSM, composite scale of morningness; SWLS, satisfaction with life scale; NS, novelty-seeking; HA, harm avoidance; RD, reward dependence; PS, persistence; SD, self-directedness; CO, cooperativeness; ST, self-transcendence.

### Correlations between CSM, SWLS, and TCI-RS data

Correlations between the CSM, SWLS, and TCI-RS response data were determined using Pearson’s correlation analysis. The CSM total score (a higher score represents a greater tendency toward morningness) was significantly correlated with age (r = 0.124, *p* < 0.05), SWLS (r = 0.271, *p* < 0.001), NS (r = −0.136, *p* < 0.05), HA (r = −0.222, *p* < 0.001), PS (r = 0.248, *p* < 0.001), SD (r = 0.223, *p* < 0.001), or CO (r = 0.194, *p* < 0.01). The SWLS total score (higher score represents greater life satisfaction) was significantly correlated with HA (r = −0.512, *p* < 0.001), PS (r = 0.376, *p* < 0.001), SD (r = 0.600, *p* < 0.001), CO (r = 0.283, *p* < 0.001), or ST (r = 0.161, *p* < 0.01).

### Moderation analysis of morningness between temperament and well-being

**Figure 1:**
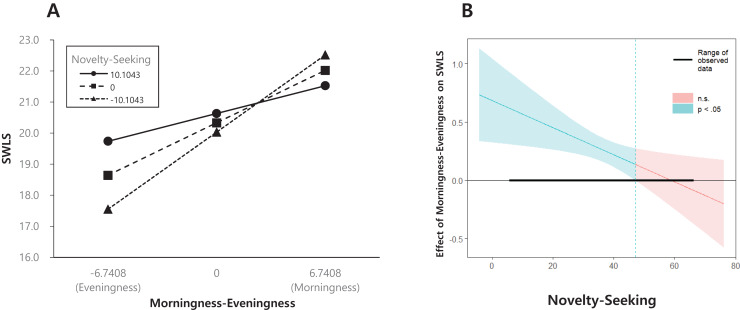
Novelty-seeking (NS) temperament as a moderator of the association between morningness and well-being. SWLS, satisfaction with life scale.

To test the first hypothesis, a moderation analysis was conducted using Hayes’ PROCESS macro (model 1) in SPSS. The first regression model, including morningness, NS temperament, and an interaction term after controlling for age and sex, proved significant, F (5, 281) = 7.507, *p* < 0.001, and explained 11.8% of the variance in well-being. The effect of NS was not significant (β = 0.030, *p* > 0.05), although the effect of morningness (β = 0.251, *p* < 0.001) and the interaction term were significant (β = −0.012, *p* < 0.05). The results showed that the positive effect of morningness on well-being increased with lower levels of NS (Fig. 1). The floodlight analysis demonstrated that the Johnson-Neyman intersection point was located at 9.695 in the NS data. Above this NS moderator value (16.028%), morningness was not related to well-being.

**Figure 2:**
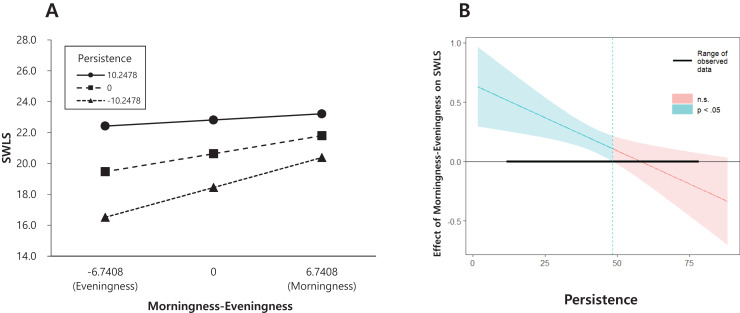
Persistence (PS) temperament as a moderator of the association between morningness and well-being. SWLS, satisfaction with life scale.

The second regression model, which included morningness, PS temperament, and an interaction term after controlling for age and sex, proved significant, F (5, 281) = 15.537, *p* < 0.001, and explained 21.7% of the variance in well-being. The effects of PS and morningness were both significant (β = 0.213, *p* < 0.001 and β = 0.173, *p* < 0.001) along with that of the interaction term (β = −0.011, *p* < 0.01). The results showed that the positive effect of morningness on well-being increased with lower levels of PS (Fig. 2). The floodlight analysis showed a Johnson-Neyman point of 5.821. Above this PS moderator value (27.526%), morningness did not impact well-being. Thus, H1, which tested the moderating effects of both NS and PS, was supported by the results.

Other regression models including HA and RD temperaments and morningness were conducted, but no interactions between temperament and morningness were observed. Therefore, the following procedures were not tested.

### Serial mediation of morningness and well-being by HA and SD

**Figure 3:**
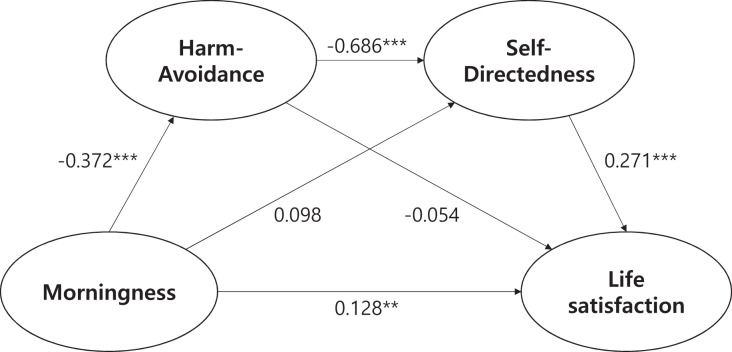
Sequential mediation of the relationship between morningness and life satisfaction by harm avoidance and self-directedness. **p < 0.01; ***p < 0.001.

To test H2, sequential mediation analysis was conducted using Hayes’ PROCESS macro (Model 6) in SPSS. The full process model, showing all path coefficients, is presented in Fig. 3. Table 2 lists the total, direct, and indirect effects. The total-effect model showed that morningness was significantly and positively associated with life satisfaction (*β* = 0.2433, *p* < 0.001). However, the effect of morningness on life satisfaction decreased (*β* = 0.1277, *p* < 0.01) after the inclusion of HA and SD in the direct-effect model. This reduction in the magnitude of the effect of morningness without a decrease in the significance level indicates partial mediation of well-being outcomes. To confirm the partial mediation effect of HA and SD, the indirect effects were scrutinized.

**Table 2:** Direct and indirect effects of morningness on life satisfaction.

Variables	β	SE	LLCI	ULCI
**Morningness →** **HA**	−0.371	0.102	−0.572	−0.171
Morningness → SD	0.098	0.066	−0.032	0.227
**Morningness** **life satisfaction**	0.128	0.044	0.041	0.215
**HA** → **SD**	−0.686	0.038	−0.760	−0.613
HA → life satisfaction	−0.054	0.037	−0.127	0.019
**SD →** **life satisfaction**	0.271	0.040	0.193	0.349
Morningness → HA → life satisfaction	0.020	0.016	−0.007	0.054
Morningness → SD → life satisfaction	0.027	0.020	−0.013	0.065
**Morningness** → **HA** → **SD** → **life satisfaction**	0.069	0.022	0.029	0.117

**Notes:**

Significant direct and indirect effects are indicated in bold.

HA, harm avoidance; SD, self-directedness; SE, standard error; LLCI, lower-limit confidence interval; ULCI, upper-limit confidence interval.

The data in Table 2 show that the indirect effects through HA or SD were insignificant (*β* = 0.0201, *β* = 0.0265; respectively). However, the indirect effect of morningness on life satisfaction through the sequential mediation of HA and SD was significant (*β* = 0.0753). The indirect effect (0.069) to total effect (0.1277) ratio in the mediation was 0.54, which showed the meaningful mediation effect. This finding provides support for the sequential mediation model in the context of personality and well-being. HA and SD failed to independently mediate the relationship between morningness and life satisfaction, while HA and SD partially mediated this relationship in sequence. Finally, the sequential mediation effects of HA and the other character dimensions of CO and ST were not supported.

## Discussion

Many studies have investigated the relationship between morningness and personality, and between personality and well-being. However, there is a paucity of research examining the direct relationship between morningness and personality, and their impact on well-being. We used the TCI-RS to study these features, which we consider is the most appropriate personality instrument for this purpose. The TCI-RS and the well-being construct share the fundamental concepts of temperament and character; in other words, well-being is influenced by innate and biological temperament factors and by mature and socio-cultural character features.

Through the use of the TCI-RS, we found the moderating effects of both NS and PS on life satisfaction according to chronotype, while HA and RD did not. The interaction effect of morningness and NS was significant when the Johnson-Neyman point was lower than 9.695 but not significant when the Johnson-Neyman point was higher than 9.695. This might mean that the effective regulation of the relationship between morningness and well-being only occurs when NS is low. Approximately 84% of the participants experienced this effect when NS was below 9.695. In the lower-NS group, morningness was associated with increased well-being, but there was no difference in well-being in the higher-NS group, regardless of chronotype.

Similarly, the interaction effect of morningness and PS was only significant when the Johnson-Neyman point was lower than 5.821. This means that the relationship between morningness and well-being was moderated only when PS was low. The significance rate of this effect was approximately 72% when PS was below 5.821. In other words, morningness in lower-PS individuals was associated with greater well-being, but there was no difference in well-being in higher-PS participants, regardless of morningness or eveningness.

In particular, the finding of moderation by NS may explain the mixed results of previous studies (Antunez, Navarro & Adan, 2014; Randler & Saliger, 2011). These studies showed that NS had a negative correlation with morningness but no relationship with well-being, which corresponds with the results of the present study. This point emphasizes the impact of the NS temperament trait as a moderator. A tendency toward high NS should be considered somewhat of an advantage for well-being, as individuals with high NS are typically passionate about new ideas and innovative activities, and might pursue avenues to well-being. Other personality variables, such as the FFM extraversion and openness items, share similarities with NS characteristics. Future studies should explore the potential moderating effects of these traits on morningness and well-being.

PS also had a moderating role in the relationship between morningness and well-being, and this factor also demonstrated a simultaneous positive correlation with morningness and well-being. Similar to the NS trait, excessive PS may not have a positive effect on well-being. The high PS score tendency showed the advantage and disadvantage effects in emotional regulation, an important subfactor of well-being (Chae et al., 2019; Cloninger et al., 2012). A person with high PS is usually industrious and determined, and could maintain strategies that are useful for achieving well-being.

According to a definition proposed by Cloninger, temperament traits such as NS and PS have a nonlinear association with character and well-being (Cloninger & Zohar, 2011; Cloninger & Zwir, 2018; Garcia et al., 2022). The level of personality variables should be considered when investigating the relationship between well-being and other variables, such as morningness. Additionally, impulsivity, a subfactor of the NS temperament, is not typically associated with well-being or adaptive emotional regulation (Schreiber, Grant & Odlaug, 2012). The association between personality sub-factors and well-being should be further explored.

Second, considering the importance of character in controlling temperament and influencing well-being, the sequential mediating effect of temperament and character was examined in the present study. The serial mediating effect of HA and SD was significant; however, NS, RD, and PS temperament traits did not show such an effect with SD. In other words, the partial mediating effect of personality on morningness and well-being was supported: morningness itself not only affects well-being, but the combination of personality variables such as HA temperament and SD character dimension exerts additional influence.

SD is an important prerequisite for character development that emphasizes protective factors, considering that HA is negatively correlated with well-being. This is consistent with previous studies (Eley et al., 2013, 2016; Kim, Lee & Lee, 2013). Individuals with high HA may focus on dangers and worries and tend to be pessimistic; however, a combination of SD and HA features could control vulnerable temperament trait (Chae, Cloninger & Lee, 2020; Lee et al., 2014) and strengthen the positive aspects thereof. The SD character dimension, widely known to represent individuals who are purposeful, competent, and self-actualizing, may influence this association (Chae, Cloninger & Lee, 2020). Therefore, joint investigations of the combination of HA and SD should be conducted. In addition, other character variables in our study, such as CO and ST, did not significantly mediate the morningness-well-being relationship, which reaffirms that SD is a prerequisite for character development among character dimensions.

Numerous studies have shown that SD is directly and linearly related to well-being. The character domain of TCI is a measure of psychological maturity, an apparent protective factor for psychopathology, and is related to biopsychological well-being (Eley et al., 2013; Moreira et al., 2015). The importance of SD in stress response has been consistently reported (Eley et al., 2013, 2016; Kim, Lee & Lee, 2013; Lee, Choi & Chae, 2017b; Sievert et al., 2016). However, to our knowledge, our study was the first to attempt to classify personality characteristics into innate and acquired influences on the relationship between morningness and well-being. As such, temperament and character, which play pivotal roles in life satisfaction and mental health, exert their influence (Chae & Lee, 2022) not only as individual traits but also through complex interactions (Eley et al., 2016).

College students usually have a circadian rhythm with an evening preference and are, therefore, presumed to experience reduced well-being. However, the results of the present study imply that the combination of the HA temperament and SD character plays a complementary role in the development of well-being, even among evening-type people. Therefore, future therapeutic interventions could focus on the promotion of SD, given the supposed stable and unchangeable nature of temperament traits. Well-being could be enhanced through the development of SD since it is a character variable that controls temperament (Chae, Cloninger & Lee, 2020; Chae & Lee, 2022; Chae et al., 2019).

There are several limitations to generalizing the findings of this study. First, although this study was performed on Korean university students with demographic features, the hypotheses should be examined in a larger sample size with a balanced age and sex distribution.

Second, our study was cross-sectional, which restricts any interpretation of causality. A well-designed, longitudinal study is required to test the temporal relationship between morningness and personality and its effects on well-being. Even though there is one longitudinal study regarding the effect of morningness on subjective well-being (Gorgol, Bullock & Stolarski, 2022), it is still too premature to draw any conclusions regarding causality. In addition, other variables which might influence the temporal relationship such as socioeconomic level and presence of psychological disorders were not asked in this study. Those variables should be considered in the future studies.

Third, well-being consists of affective (*e.g*., positive and negative affect) and/or non-affective components (*e.g*., life satisfaction, perceived social support, or perceived health). Therefore, life satisfaction is just a part of well-being, even though we used both terms interchangeably in current study, as have other studies (Drezno, Stolarski & Matthews, 2019; Nima et al., 2020). Further research would benefit from including emotional component, such as positive and negative affect, in research on well-being.

Fourth, there is a report emphasizing the importance of circadian amplitude when considering the relationship between well-being and morningness-eveningness in college students (Diaz-Morales et al., 2017). Measuring the circadian amplitude in addition to morningness-eveningness is recommended for future studies in order to investigate the factors that influence well-being. Finally, we used one-time self-report questionnaires, so the potential for evaluative bias in the results cannot be discounted. An experience sampling method is recommended for multiple assessments of variation at several different times of the day.

In summary, this study explored how the interplay between morningness and temperament influence well-being by examining moderating effect of temperament on the relationship between morningness and well-being. Furthermore, this study provided significant insight into the influence of temperament and character dimensions on the effects of the relationship between morningness and well-being (Adan et al., 2009; Rio-Martinez et al., 2020) by investigating sequential mediating effect of temperament and character based on the Cloninger’s biopsychosocial model. Consequently, this study might serve as a reminder of the significance of SD in terms of protective factor and promoter of well-being. In this regards, we also suggest the use of a person-centered approach (Garcia et al., 2022; Moreira, Inman & Cloninger, 2021; Wong & Cloninger, 2010) in future research for a more comprehensive understanding of within-individual structures and processes.

## Supplemental Information

10.7717/peerj.15861/supp-1 Supplemental Information 1 Raw TCI Wellbeing Data.

## Additional Information and Declarations

### Data Availability

The following information was supplied regarding data availability: Raw data are available as a Supplemental File.
